# Linguistic Prediction in Autism Spectrum Disorder

**DOI:** 10.3390/brainsci15020175

**Published:** 2025-02-10

**Authors:** Aimee O’Shea, Paul E. Engelhardt

**Affiliations:** School of Psychology, University of East Anglia, Norwich Research Park, Norwich NR4 7TJ, UK; a.oshea@uea.ac.uk

**Keywords:** autism spectrum disorder, linguistic prediction, cloze probability, voice onset time

## Abstract

Background: Autism spectrum disorder has been argued to involve impairments in domain-general predictive abilities. There is strong evidence that individuals with ASD have trouble navigating the dynamic world due to an inability to predict the outcomes of particular events. There is also evidence that this is apparent across the diagnostic criteria of ASD and common among correlates of ASD. However, the question remains as to whether this impairment in predictive abilities is domain-specific or domain-general, with little research investigating prediction in linguistic measures. Methods: The current study investigated whether individuals with ASD showed atypicalities in linguistic prediction using a cloze probability task. In Experiment 1, 33 individuals with ASD were compared to 64 typically developing individuals in an offline cloze task. Results: There was no significant effect of an ASD diagnosis on the cloze probability. However, individuals with higher levels of autistic traits were significantly more likely to produce lower-probability (non-modal) cloze responses. In Experiment 2, 19 individuals with ASD were compared to 22 typically developing individuals in a lab-based cloze task, in which we also measured the reaction times to begin speaking (i.e., voice onset time). The results showed that individuals with ASD had significantly slower reaction times (~200 ms) but, similarly to Experiment 1, did not show differences in the cloze probability of the responses produced. Conclusions: We conclude that individuals with ASD do show inefficiency in linguistic prediction, as well as indicating which ASD traits most strongly correlate with these inefficiencies.

## 1. Introduction

Autism spectrum disorder (ASD) is a neurodevelopmental disorder characterised by persistent deficits in social communication and interaction, special interests, and repetitive patterns of behaviour that tend to manifest in early development [1]. Individuals on the autism spectrum tend to have problems throughout their lifespan with language abilities, with some pertaining to the universal impairments in pragmatic language [2] but others being attributed to atypical structural language skills, which are considerably more varied across the spectrum [3]. Linguistic prediction is an area of psycholinguistics that has been under-studied in individuals on the autism spectrum but, in neurotypical individuals, is thought to play a key part in the comprehension of language, ultimately facilitating communication [4]. The current study sought to explore whether individuals on the autism spectrum differed from neurotypical individuals in their linguistic prediction abilities.

### 1.1. Theory of Prediction in Autism

There is currently no universal theory that accounts for or fully encompasses the phenotype that is observed in individuals on the autism spectrum. ASD is known to be a highly heterogeneous disorder, and the main symptomologies that constitute the diagnostic criteria of the disorder are phenotypically dissimilar in nature. This has provoked questions as to how they have been classified to ultimately represent ASD and whether there is one underlying cognitive mechanism that may explain all of them.

The hypothesis of predictive impairment in autism (PIA) suggests that individuals with ASD experience atypicalities in predictive processing [5,6,7]. In the brain, estimations are made on the probability of transitioning from an antecedent to a consequence. For a typical individual, this estimation is based on “coded” learning experiences. When the individual experiences the same antecedent again, the brain is able to predict the consequence based on how it was previously coded. The PIA hypothesis assumes that individuals on the autism spectrum may make significant prediction errors, meaning that this system operates in a fundamentally different way to that in typical individuals [6]. According to the theory, these errors can account for the symptoms of ASD that constitute its diverse phenotypic profile.

In order for the PIA hypothesis to have merit, it would need to account for both key markers of ASD: deficits in social communication and interaction and restricted and repetitive behaviours [1]. Deficits in social communication and interaction may occur as a result of the unpredictability of dynamic social environments, where individuals on the autism spectrum experience atypical processing of social rules and cues; therefore, navigating these kinds of situations becomes increasingly difficult, resulting in avoidance [6,7]. If this avoidance is manifested in early childhood, then dysfunctional social skills may arise, further fostering the avoidance of such experiences. Restricted and repetitive behaviours may represent an aversion to new, unpredictable activities and experiences, which cause discomfort to individuals on the autism spectrum, leading to a clinically significant reliance on rituals and routines [5,6]. Other correlates of autism can be at least partially explained by atypicalities in predictive processing, such as the links between ASD and Theory of Mind, the observed sensory hypersensitivities, and the high prevalence of anxiety in individuals on the autism spectrum [6]. There is a large body of evidence exploring the predictive coding errors of individuals on the autism spectrum, across experimental paradigms and modalities, including spontaneous gaze [8,9], emotion prediction [10], auditory prediction [11], and social expectations [12] (for a review, see [13]).

### 1.2. Linguistic Prediction

If there were domain-general predictive impairments in individuals on the autism spectrum, then we would expect this impairment to be evident in linguistic prediction tasks. Linguistic prediction is thought to play a key role in the comprehension of language by contributing to conversational skills and correct conduct, ultimately aiding the efficiency of communication [14,15,16]. The basic assumption is that the comprehension system predicts a linguistic unit before receiving the bottom-up input: the speaker does not wait to hear the whole stimulus, but makes an estimation about the following word based on the characteristics of the preceding words [17,18]. For example, if a speaker said the sentence, “I am going to eat the…”, the listener would be reasonable to predict that, based on the lexical information provided (i.e., the verb eat), the speaker was going to consume an edible object, rather than an inedible object (e.g., scissors) [19]. Thus, the listener would expect a food-related noun (an edible object) to occur next in the string (e.g., sandwich). Problems with linguistic prediction have an impact on language comprehension and are subsequently suspected to play a fundamental role in language acquisition [20]. This has been evident across studies with neurotypical individuals, who experience processing difficulties, such as longer reading times, when ambiguous bottom-up input is incongruent to their top-down “coded” expectations, e.g., [21,22,23,24,25,26]. On the other hand, it is common for individuals with poorer literacy skills to face problems with linguistic prediction, which is evident in lower-level readers, individuals with dyslexia, and second language (L2) processing [27,28,29,30,31,32].

Given that individuals on the autism spectrum show an atypical language profile, it would be reasonable to question whether issues with linguistic prediction occur in this population. However, given the body of evidence surrounding linguistic prediction in neurotypical individuals, these associations have the potential to be bi-directional. If atypical predictive processes occur in language, as they occur for other aspects of the ASD phenotypic profile (in accordance with the PIA hypothesis [6]), this may result in poorer language comprehension. This would ultimately affect conversational skills, potentially contributing to deficits in social communication and interaction, which are at the centre of the ASD diagnosis, and would provide support for a domain-general predictive impairment in ASD. On the other hand, as poorer language abilities tend to result in issues in effectively utilising predictive processes, it may be the case that the results found concerning atypical linguistic prediction abilities in individuals on the autism spectrum instead represent evidence for a broader language issue in ASD, rather than a difficulty with prediction itself. This would then contribute to the trend in ASD research of language abilities being a notable confounder. However, we believe that the latter argument can be refuted by controlling for language abilities in prediction research, performing fairer and more meaningful comparisons between individuals on the autism spectrum and typically developing control participants.

### 1.3. Is There Evidence for Impaired Linguistic Prediction in ASD?

There is some evidence for impaired linguistic prediction in individuals on the autism spectrum. Prescott et al. [33] investigated predictive language processing using an eye-tracking paradigm, finding that, whilst autistic children engaged in similar predictive processes by directing anticipatory eye movements to target nouns, the effect size was larger in neurotypical individuals. Huettig et al. [34] similarly employed an eye-tracking paradigm to investigate eye movements in children in a look-and-listen task. Participants were presented with spoken sentence stems and subsequently shown two objects: one that was semantically appropriate and one unrelated distractor. The results showed that neurotypical children successfully utilised predictive processes by showing preferential eye movements to the target object, whereas this was significantly reduced in children on the autism spectrum. Zhao et al. [35] investigated linguistic prediction in individuals on the autism spectrum using a cloze probability task, where participants were tasked with completing a sentence stem with the word that they thought “best fit” the sentence. Zhao et al. [35] found that individuals on the autism spectrum produced significantly less frequent words than neurotypical participants. These studies provide evidence for impaired linguistic prediction in individuals on the autism spectrum. However, it is important to note that some of these results were modulated by language abilities. Individuals on the autism spectrum in Prescott et al. [33] had significantly lower scores on a measure of expressive communication. Likewise, individuals on the autism spectrum in Zhao et al. [35] had significantly lower scores on a vocabulary test. This is particularly problematic when, as pointed out by Andreou and Lymperopoulou [36], language production and wider vocabulary knowledge are key factors in the ability to successfully engage predictive processes in language comprehension.

Other evidence attests to intact linguistic prediction in individuals on the autism spectrum. Bavin, Kidd, Prendergast, and Baker [37] used an eye-tracking task to investigate the processing of sentences containing bias and neutral verbs in children with and without ASD. The results showed that children on the autism spectrum showed preferential eye movements to the biasing verbs compared to the neutral verbs, and there were no significant differences between groups, providing evidence that predictive processes were being utilised to the same effect as they were for typically developing control participants. However, Bavin et al. [37] found that this process was significantly slower for the ASD group. Zhou, Zhan, and Ma [38] also used the visual world paradigm to test ASD and typically developing Mandarin-speaking children. Zhou et al. [38] found that children on the autism spectrum showed predictive eye movements to target areas during real-time sentence comprehension using neutral and biased verbs, and this was equally as quick as it was for typically developing children. However, children on the autism spectrum exhibited fewer fixations back to the target object after the initial presentation. Zhou et al. [38] suggested that this was due to problems in maintaining visual attention, which is common in ASD participants [39,40]. Utilising a different modality, Brennan, Lajiness-O’Neill, Bowyer Kovelman, and Hale [41] used MEG to investigate the mechanisms underlying behavioural outcomes from eye-tracking data. They found that children on the autism spectrum were able to engage in predictive sentence comprehension, whilst listening to an audiobook story, shown by neuromagnetic signals that quantified prediction in terms of surprisal, and these results were not affected by verbal abilities, which were significantly lower in the ASD group.

Based on this evidence, a few points are worth contemplating. Firstly, the breadth of studies investigating linguistic prediction in ASD is considerably limited and varied, with no universal consensus on whether prediction in this population is impaired in comparison to typically developing individuals. Some studies finding evidence for impaired linguistic prediction in individuals on the autism spectrum are weakened by a failure to control for differences in language abilities [33,35]. Those finding evidence for intact, or at least comparable, linguistic prediction in ASD note some subtle differences, which can be attributed to longer processing times in individuals on the autism spectrum (e.g., [37]). This is consistent with other evidence highlighting similar comprehension accuracy for behavioural measures in individuals on the autism spectrum, but differences in the processing of stimuli when analysing online data [42]. It is therefore important to consider whether predictive coding errors are ultimately defined by inaccuracies made in the final comprehension of the linguistic input or whether the prediction process itself is temporally bound and it is this variable that is indicative of an overall impairment in this domain (or whether this reflects a prediction inefficiency rather than impairment). We can therefore assume, based on this, that there is perhaps not an impairment in predictive abilities for individuals on the autism spectrum, but that their predictive abilities are potentially atypical, questioning the PIA hypothesis, which adopts the stance that there is a broad domain-general predictive impairment in ASD that is consistent across experimental paradigms. This is something that should be explored in more depth.

### 1.4. The Present Study

We believe that investigating the mechanisms that may be at play that could potentially provide a better understanding of the diverse ASD phenotype is valuable because it can guide well-informed interventions, ultimately laying the foundation for better adjustments for individuals in this population. There is currently no strong evidence for impaired linguistic prediction in individuals on the autism spectrum, yet the PIA hypothesis is still prominent in discussions concerning ASD phenotypes. We aimed to investigate whether linguistic prediction was impaired in individuals on the autism spectrum.

Linguistic prediction has been assessed in many psycholinguistic studies using a cloze probability task [27,35,43,44]. The purpose of this task is to investigate how individuals predict the end of a sentence [45]. Sentence stems with a cloze probability of less than 0.5 are classed as low-constraint sentences and, therefore, do not afford much (or very little) prediction. Those with a cloze probability of 0.5 or above are classed as high-constraint sentences [44]. In short, high-constraint sentences are more predictable than low-constraint sentences, as evidenced by a higher mean cloze probability. In the current study, we focused our analysis only on high-constraint sentences. Comprehension for sentences is much more difficult when the sentence has a lower predictability. Therefore, the cloze probability has traditionally been the gold-standard measure of prediction in psycholinguistics.

To our knowledge, there is only one study [35] that has utilised a cloze probability task in individuals on the autism spectrum. Given that these kinds of tasks are an irrefutable measure of linguistic prediction and are not bound by the limitations of visual attention that are common in other linguistic prediction tasks used in this area of research [38,41], we believe that it is particularly valuable to measure cloze probabilities in this population. The study by Zhao et al. [35] found significant differences in the number of higher-cloze-probability responses produced between ASD participants and typically developing control participants. However, these participants were not matched in terms of language abilities, the importance of which is reviewed above. We conducted two studies. The first was an online (internet-based) study to determine whether individuals on the autism spectrum differed compared to typically developing individuals in their overall cloze probability responses. This served as an initial study to determine whether there were any obvious disparities in predictive abilities in a low-demand task with no experimenter present. The second study replicated the first in a lab setting, using the auditory modality, and the rationale for doing so was to measure the reaction times to begin speaking. Thus, Experiment 2 also had an online measure of processing. In the second experiment, we also assessed vocabulary abilities in all participants. The current study was guided by three broad research questions.

1. Is there evidence for an impairment in linguistic prediction among individuals with ASD?
2. Is this impairment shown in the overall lower cloze probability of the words produced or by slower processing times?
3. Are impairments in linguistic prediction apparent when controlling for differences in language abilities (i.e., a vocabulary measure)?

## 2. Experiment 1

In this experiment, we compared ASD participants to typically developing controls in an online study. Participants read sentence stems and completed the sentence by typing the word that they thought was the “best” or most natural continuation. We hypothesised that individuals with ASD would produce significantly lower-cloze-probability responses [35] and would do so across the critical high-constraint items and that the same pattern would hold for both modal and non-modal responses. In a second set of analyses, we examined the correlations between the cloze probabilities and Autism Quotient (AQ) scores (as measures of traits of autism) (see Materials, Section 2.1.2). The second set of analyses examined ASD traits via a continuum approach. However, the direction of the hypothesis was the same: higher ASD traits (higher AQ scores) would be negatively related to the cloze probability. Finally, we conducted two sets of regression analyses to determine whether (1) significant effects held when age and gender were included in the statistical models and (2) which of the ASD symptom clusters linguistic prediction was more related to.

### 2.1. Methods

#### 2.1.1. Participants

The target sample for this study consisted of individuals with a university-level education, who were recruited via social media platforms, with a formal diagnosis of ASD. These participants were all high-functioning individuals with autism. Typically developing individuals who did not have a formal diagnosis of ASD were also recruited as controls. In total, 109 participants took part in the study. All participants were native speakers of British English. Participant exclusion criteria included an age below 18 or above 60 years, which resulted in the removal of seven control participants. We also examined the Autism Quotient (AQ) score and trimmed outliers at (1) the upper end of the distribution for controls (i.e., elimination of false negatives) and (2) the lower end of the distribution for the ASD group. This led to the further removal of two participants from the control group and three participants from the ASD group. Thus, 12 participants were excluded in total. Note that the gender “significance” (Table 1) was calculated by excluding the “other” or “unreported” participants.

ASD Group. Thirty-three individuals had a self-reported diagnosis of ASD. The age range of the participants with ASD was 18–59 years (M = 30.76, SD = 12.31). There were five males, 21 females, and seven other/unreported.

Control Group. There were 64 control participants. The age range of the control group participants was 18–60 years (M = 34.03, SD = 12.30). There were 15 males and 49 females. Table 1 shows the means for the demographic variables.

Recruitment Strategy. Participants were recruited via advertisements on social media, mainly through Facebook. (This study took place during the height of the worldwide COVID-19 pandemic.) The ASD group was recruited on pages designed specifically for individuals with ASD to take part in research. There was no incentive given other than to help the programme of research. Participants were given the researchers’ contact details in the initial advertisement so that they could ask any questions before taking part. The link provided in the advertisement took the participants directly to Qualtrics, where the study was completed.

#### 2.1.2. Materials

Autism Spectrum Quotient. The AQ is a self-report measure of autistic traits [46], consisting of 50 items assessing ASD symptomology in five areas (social skills, attention switching, attention to detail, communication, and imagination). Answers are given on a four-point Likert scale with the options “Definitely Agree”, “Slightly Agree”, “Slightly Disagree”, and “Definitely Disagree”. Scores on the AQ are summed and can range from 0 to 50, with a higher score indicating a higher level of autistic traits. For the purposes of the current study, the subscales of the AQ were also summed, providing a total AQ score. Descriptive statistics for the total AQ score, as well as the subscale scores, across both the ASD group and the control group are reported in Table 1. The Cronbach’s alpha of the AQ for the current study was α = 0.90, demonstrating high internal consistency.

Adult Repetitive Behaviours Questionnaire 2 (ARBQ-2A) [47]. The ARBQ is a self-report measure of repetitive behaviour in adults, consisting of 20 items assessing two domains: insistence on sameness and repetitive motor behaviours. The questionnaire is separated into five sections.

For sections 1–4, answers are given on a three-point Likert scale, with the options “never or rarely” (scoring 1), “mild or occasional” (scoring 2), and “marked or notable” (scoring 3). For section 5, the answer is given on a three-point Likert scale, with the options ‘a range of different and flexible self-chosen activities’ (scoring 1), ‘some varied and flexible interests but commonly choose the same activities’ (scoring 2), and ‘almost always choose from a restricted range of repetitive activities’ (scoring 3). Answers are summed and scores can range from 20 to 60. A higher score indicates that the person has a higher level of repetitive behaviours.

Cloze Probability Task. The sentence stems used for the current study were derived from materials used in [45] (see Table 2 for examples). From the 152 sentence stems, 100 were randomly selected for use in the current experiment (50—high constraint and 50—low constraint). (Low- vs. high-constraint items were determined by the most frequent words provided for each stem. Items with the highest word <0.50 were classified as low constraint, and items with the highest word >0.50 were classified as high constraint. Low-constraint sentences served as filler trials.) There were an additional three items, which were used as practice trials.

#### 2.1.3. Design and Procedure

The design of the study consisted of a single independent variable, group (ASD vs. control), which was between participants. The main dependent variable was the mean cloze probability for the 50 high-constraint items. The calculation of the cloze probability followed the typical procedure. For example, if there are 33 out of 100 people who complete the sentence stem “the airplane went into a ____” with the word “spin”, this would make the cloze probability of the sentence 0.33. The cloze probabilities for our items were determined based on the responses provided by all participants in the current study (i.e., we calculated the cloze probabilities by assessing the responses to each item across the entire sample of participants). We then calculated the average cloze probability for each participant across the set of 50 critical (high-constraint) items. Next, we classified the responses based on whether they were modal or non-modal [44]. Modal responses are the most frequent words provided for each stem. Returning to the example above, if spin was the most frequent word produced, then this response would be classed as “modal”. All other responses would be classed as non-modal. By this classification scheme, the responses are divided into two categories, and, by definition, the non-modal responses have lower cloze probabilities compared to the modal responses. We again calculated the mean cloze probability by averaging across the items for each participant. Thus, each participant in the study had (1) an overall cloze probability mean, (2) a modal cloze probability mean, and (3) a non-modal cloze probability mean. These three scores were the dependent variables in the analysis.

If participants were interested in taking part in the study, they were asked to click the link, which took them directly to Qualtrics. Participants first had the opportunity to read an information sheet and then they clicked a consent box. Participants then proceeded to the study. They first completed a short demographic questionnaire, followed by the AQ [46]. Following these, they completed the repetitive behaviours questionnaire (participants also completed two unrelated anxiety questionnaires). This took approximately 15 min. Participants were then instructed to click forward to the experimental cloze probability task. For the cloze task, participants were instructed to read an incomplete sentence stem and type the word that they thought “best fit” the sentence. In each sentence, the final word of the sentence was missing. In total, there were 100 sentences (50 critical high-constraint items and 50 low-constraint filler items). This took approximately 15 min to complete. After the experimental task, participants were shown the debrief. The anonymous data were stored separately from the participants’ names. The study was approved by the School of Psychology Research Ethics Committee at the University of East Anglia (UK) (ETH2223-0946 (27 November 2022)). Informed consent was obtained from all participants before carrying out the study, and all were debriefed at the end of the study.

### 2.2. Results

#### 2.2.1. Data Analysis Plan and Data Preparation

The data analysis plan had two main components. The first was to compare the groups (ASD vs. control) regarding the mean cloze probability for the full set of critical items, using an independent-samples *t*-test. We also examined the mean of “modal” and the mean of “non-modal” responses, again using independent-samples *t*-tests. Modal responses had higher mean cloze probabilities compared to non-modal responses. The second component of the analysis examined the correlations between age, gender, and the total AQ scores and the dependent variables in the cloze task. We followed up the correlations with two sets of multiple regressions. The first set of multiple regressions examined the total AQ scores, age, and gender regressed onto the cloze probabilities. The second set of multiple regressions examined the AQ communication subscale, the ARBQ repetitive behaviours measure, age, and gender. The purpose of the second set of analyses was to ascertain whether prediction effects were associated with the two main (DSM) diagnostic symptom clusters of ASD. Prior to the statistical analyses, the dependent variables were assessed for outliers and for a normal distribution (i.e., that the skew value was less than two times the standard error). For this experiment, the standard error was 0.246, and the skew was less than ±0.418. Missing data (due to incomplete responses) constituted less than one percent of the trials (i.e., 0.7%).

#### 2.2.2. Independent-Samples *t*-Tests

Independent-samples *t*-tests were performed to determine whether the groups differed in the overall cloze probability, modal cloze probability, and non-modal cloze probability (see Table 3). The results showed no significant differences. Thus, there was no evidence of group differences in terms of the cloze probability (i.e., linguistic prediction).

#### 2.2.3. Autism Traits

The correlations between the demographic variables, AQ scores, and repetitive behaviours are presented in Table 4. The results showed that gender correlated with the overall cloze probability, whereas age correlated with the modal cloze probability. (The positive gender correlations indicate that females have higher-cloze-probability responses). Autism traits, measured by the AQ total scores, did not correlate with any of the dependent variables. AQ communication and repetitive behaviours showed several significant correlations; specifically, communication was significantly related to the overall cloze probability, and repetitive behaviours was correlated with all three dependent variables. The patterns across all ASD measures indicated negative relationships for the overall and non-modal cloze probabilities (i.e., higher levels of ASD traits corresponded with lower/worse prediction) and positive relationships for the modal cloze probability (i.e., higher levels of ASD traits corresponded with higher/better prediction). These trends demonstrate that individuals with higher ASD traits showed better prediction (or a higher likelihood of producing modal responses). However, in cases in which the modal response was not produced, higher levels of ASD traits were associated with worse prediction. In short, in situations where the modal prediction did not run through, high-ASD-trait individuals produced more lower-cloze-probability responses. We interpret these trends (across the dependent variables) as showing that the tendency to produce fewer non-modal responses was stronger than the tendency to produce more modal responses, as shown by the negative relationship across the full set of critical items (i.e., overall cloze probabilities). Note that our interpretation of these trends is independent of statistical significance, as exactly half of the examined correlations were not significant.

The ASD group did not differ from the control group in this study based on age, but they did significantly differ in gender. In order to follow up on the significant findings with respect to ASD traits, we ran an initial set of three regressions, which included the total AQ score, age, and gender as predictors. The results of these regressions are shown in Table 5, regressions 1–3. There were marginally significant effects of the AQ scores on both the overall cloze probability and modal cloze probability. There was a significant effect of the AQ scores on the non-modal cloze probability. Importantly, these findings held even when age and gender were included in the regression models. The pattern of the regression coefficients showed a positive effect on the modal cloze probability and negative effects on the overall cloze probability and non-modal cloze probability, being similar to the bivariate correlations.

In the second set of regressions (see Table 5, regressions 4–6), we examined the AQ communication subscale and the ARBQ questionnaire. (Note that we did not include the AQ total scores in this analysis due to the strong correlation between it and AQ communication.) The reason for the second set of analyses was to determine whether the prediction differences were related to the two main DSM diagnostic criteria of ASD, an issue initially raised in the Introduction. The results showed that AQ communication was not related to any of the dependent variables when included in a multiple regression and that repetitive behaviours were significantly related to only modal responses. Based on these findings, we conclude that linguistic prediction is more strongly related to the restricted interests/repetitive behaviour symptom cluster, although more research is needed to definitively confirm this conclusion.

### 2.3. Discussion

The main group results with respect to prediction in this experiment were not significant. We did not observe significant group differences in any of three different cloze probability measures. In fact, the proportions were nearly identical for the two groups. This suggests that individuals with ASD do not experience impairments in linguistic prediction. However, when we analysed ASD traits as a linear variable (i.e., using the AQ total scores), there were two marginally significant results and one significant result.

The pattern of the results showed that the AQ total scores were marginally (negatively) related to the overall cloze probability and significantly (negatively) related to the non-modal cloze probability. This suggests that individuals with higher levels of ASD traits showed lower overall cloze probability responses and lower mean cloze probability responses for non-modal responses. In contrast, the pattern was reversed for modal cloze probability responses (i.e., higher levels of ASD traits corresponded to higher-cloze-probability responses when the modal response was given). This reversed pattern was also only marginally significant. These positive/negative patterns suggest that high-AQ individuals were more likely to produce modal responses compared to low-AQ individuals, which contradicts the hypothesis of this study. However, there were significant differences for non-modal responses, and this significant finding was stronger than the “reversed” pattern observed for modal responses. This is evidenced by the marginal/significant divergence and also by the fact that the overall cloze probabilities were negatively related to the AQ scores. Again, this result showed only marginally significant differences. What this pattern of results suggests is that, in situations in which the modal “prediction” is not activated (and therefore not produced), participants with high-AQ scores were actually more likely to produce a significantly lower-probability response, which is indicative of little to no prediction. In other words, when the key word/completion was not activated, participants with high levels of ASD traits struggled much more than those with low levels of ASD traits. The effect sizes of the AQ scores in the multiple regressions were small to medium. Ultimately, what these results suggest is that there is not strong evidence for impairments in linguistic prediction in ASD. When ASD traits were examined using a linear variable, which naturally have more predictive power potential, there are, at the very best, small to medium effects on linguistic prediction.

With respect to the relationship between prediction and individual symptom clusters, we observed that AQ “communication” scores were significantly correlated with the overall cloze probability, and repetitive behaviours were significantly correlated with all three dependent variables. However, our second set of regression analyses showed that only repetitive behaviours was significantly related to modal responses. Based on this finding, and the results of the correlations, it would seem that differences in linguistic prediction are more closely related to restricted interests/repetitive behaviours, as compared to social interactions and communication. We return to this issue in Section 4.

## 3. Experiment 2

One limitation of Experiment 1 was that it did not have an online measure of processing. Thus, in the second experiment, we conducted a lab-based study in which participants heard the sentence stems and had to produce (by speech rather than typing) the word that they thought best fit. We used the same items from Experiment 1. Thus, this study was essentially a replication of Experiment 1, except that it used the auditory modality and it assessed not only the cloze probability but also the reaction times (voice onset time) for participants to begin speaking. Furthermore, based on [37], we hypothesised that individuals with ASD would show significantly slower reaction times compared to the controls (i.e., in the online measure). Based on the results from Experiment 1, we did not expect to find significant differences in the cloze probabilities (i.e., in the offline measure). In this experiment, we also assessed vocabulary as a key measure of linguistic abilities. In order to compare the results across the experiments, we also conducted the same correlation and regression analyses as in Experiment 1.

### 3.1. Methods

#### 3.1.1. Participants

Forty-one undergraduate University of East Anglia students were recruited for this study. There were 19 with ASD and 22 typically developing individuals who served as controls (see Table 6). All were thus currently engaged in university-level education. All ASD participants verified that they had undergone a prior diagnostic assessment for autism. All were native speakers of English, with normal or corrected-to-normal vision. Participants were compensated for their time, either with participation credits or with a GBP 7 Amazon voucher.

#### 3.1.2. Materials

Autism Spectrum Quotient. Same as Experiment 1. Descriptive statistics for the total AQ and subscales across both groups are shown in Table 6.

Peabody Picture Vocabulary Test-4 (PPVT-4). The PPVT-4 [48] is a test to measure receptive vocabulary. The researcher presented a target word and participants chose an image from four different image options. The reliability for Form A is between 0.89 and 0.97.

Cloze Probability Task. The audio files were recorded by the first author (a native British English speaker) using the Audacity software (Audacity 3.7.1.). Each sentence was recorded with an anomalous word in the sentence’s final position. This ensured no coarticulation effects between the final and penultimate words in the sentence. The anomalous word was then digitally removed.

#### 3.1.3. Design and Procedure

There was a single independent variable (group: control vs. ASD), which was between subjects. We assessed the mean cloze probability of the responses provided for each of the 50 high-constraint sentences. We then coded the responses into modal and non-modal and computed means for both categories. The cloze probabilities were one of the dependent variables of the study and were calculated in the same way as in Experiment 1. The second dependent variable was the reaction time (RT), which was defined as the time from the end of the last word in the recorded sentence to the voice onset time of the participant’s response (i.e., from the end of the recording to when the participant began speaking). Reaction times, likewise, were considered for all critical items and then for modal and non-modal responses separately.

Before the start, participants were given an information sheet outlining the study. The researcher answered any questions. All participants then gave written informed consent. Participants first filled out a demographic questionnaire and then the AQ [46]. They then completed the PPVT. These tasks took approximately 20 min. (Participants in this study also completed an unrelated eye-tracking task and completed the talking sections of the ADOS-2 [49]).

For the prediction task, participants were instructed to read the instructions, which indicated that they would hear a sentence in which the final word was missing, and their task was to complete it as naturally as possible. There were three practice trials and 100 experimental trials. If they could not think of a word, participants could respond, “I don’t know”. “I don’t know” responses were excluded from all analyses. Participants pressed the space bar after each response, and the prediction task took approximately 15 min. All participants were given an information sheet and consent form. After providing consent, participants completed the various questionnaires and the experimental task, and they were debriefed following the study. The study was approved by the School of Psychology Research Ethics Committee at the University of East Anglia (UK) (ETH2223-0946 (27 November 2022)). Informed consent was obtained from all participants before carrying out the study, and all were debriefed at the end of the study.

### 3.2. Results

#### 3.2.1. Data Analysis Plan and Data Preparation

The data analysis plan had three main components. First, because there is a linear relationship between the cloze probability of words produced and the reaction time [44], we assessed the correlation between the cloze probability and the reaction time. This allowed us to compare the results from the current study to prior studies examining cloze probabilities and reaction times, and to ensure that our task was operating as intended. The second component (focusing on the hypotheses of the study) was to compare the groups (ASD vs. control) in terms of the mean cloze probabilities and mean reaction times, using independent-samples *t*-tests. The main prediction was that the groups would not differ in the cloze probability (replication of Experiment 1), but they would differ in the mean reaction time (ASD > control). Given the relationship among the cloze probability and reaction time, we followed up the reaction time *t*-test analyses with one-way ANCOVAs, in which the group was a fixed effect and the cloze probability was a covariate. These follow-up ANCOVA analyses allowed us to examine whether there were significant group effects on the reaction time, after removing the variance due to the cloze probability of the words produced. If the group effect remained significant, this suggested that the reaction time differences were not due to differences in the cloze probability of the word produced; essentially, we sought to determine whether the group exerted an effect beyond the typical cloze–RT relationship.

Prior to the inferential analyses, outliers were assessed and we ensured that the dependent variables were normally distributed (i.e., that the skew was less than two times the standard error). For the cloze probabilities, the standard error was 0.369, and the skew statistic was less than ±0.534. The reaction time data were skewed and so we applied a logarithm transformation, which was then used in the inferential statistical analyses. In addition, there were two reaction time outliers (2.8 SDs and 3.1 SDs from the mean). Both data points occurred in a control participant, and the outlying values were replaced with the mean of that condition. Missing data (due to incomprehensible or inaudible responses or “I don’t know” responses) constituted approximately 4% of the data. There were 18 trials in which a participant responded “I don’t know”; of these, 15 were produced by a control participant and 3 were produced by a participant with ASD.

#### 3.2.2. Cloze Probability–Reaction Time Relationship

**Figure 1:**
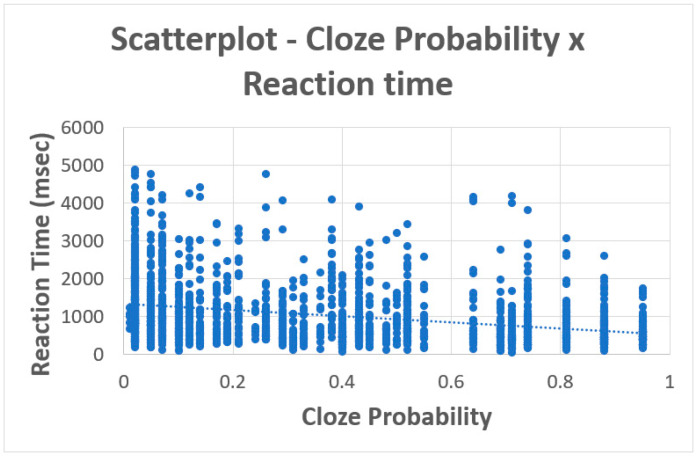
Scatterplot showing the relationship between cloze probabilities and reaction time.

The correlation between the cloze probability and reaction time was *r* (2037) = −0.31, *p* < 0.001 (see Figure 1). This is similar to what has been reported in prior studies [43,44].

#### 3.2.3. Independent-Samples *t*-Tests

We began the analysis by assessing group differences in the cloze probability and reaction times on the 50 critical high-constraint items. We also calculated the mean reaction times and cloze probabilities for the modal and non-modal responses produced. The results from these analyses are provided in Table 7.

We observed almost identical results for the cloze probabilities as compared to Experiment 1. The only differences were for the controls in the modal and non-modal responses, with a mean difference of 0.02 in both cases. Essentially, this experiment achieved the virtually perfect replication of the offline results of Experiment 1. The results for the reaction times showed two significant differences. The ASD participants had significantly slower reaction times for the overall cloze probabilities and for modal responses. The significant differences were 221 ms and 230 ms, respectively. The non-significant reaction time difference (non-modal) was 166 ms. This shows that participants with ASD were slower to issue their responses for all three analyses, two of which achieved statistical significance.

When the cloze probability was included as a covariate in a one-way ANOVA (group as IV and reaction time as DV), the main effect of the group on the overall cloze probability remained significant (*p* = 0.05). Similarly, when the cloze probability was included in the model for the modal cloze probability, the main effect of the group was also significant (*p* = 0.034). This shows that the reaction time effects held even when the reaction time × cloze probability relationship was accounted for.

#### 3.2.4. Autism Traits

For the demographic variables and cloze probabilities, there was only one significant correlation between age and the non-modal cloze probability (see Table 8), which showed that older participants produced lower-probability responses (i.e., less prediction). For the demographic variables and reaction times, there were no significant correlations (all *p*’s > 0.24). However, this study had a substantially smaller sample as compared to Experiment 1. Despite the power difference between the studies, we again report the correlations between the AQ total scores and AQ communication subscale and the cloze probabilities (see Table 8). These were included to enable a comparison to the results of Experiment 1. There was a fairly high degree of similarity between the two studies, expect for the results of the modal cloze probability. Recall that, in Experiment 1, the pattern of results was such that there were consistent negative correlations between the AQ total and overall cloze probability and non-modal cloze probability and a generally consistent pattern of positive correlations between the AQ total and modal cloze probability. In contrast, the patterns in Experiment 2 showed consistently negative correlations for the modal cloze probability. Thus, in this experiment, those with higher levels of ASD traits showed across-the-board weakness in prediction. It is important to note that the correlations for Experiment 2 were not significant, but there was a clear difference in numerical trends, specifically concerning positive/negative results.

Four further points are worth mentioning about the results. First, when age, gender, and the AQ total scores were regressed onto the cloze probabilities, there were no significant results. Second, the AQ communication subscale showed more than twice as strong a correlation with the overall cloze probability as compared to the AQ total, with a stronger correlation than in Experiment 1. When communication was included in a regression model with age, gender, and PPVT, it was a significant predictor of the overall cloze probability (see Table 9). Thus, in this experiment, AQ communication was more related to the cloze probability, as compared to the results in Experiment 1. The third point is that the reaction times did not significantly correlate with the AQ total (all *p*’s > 0.17), although this result comes with the caveat that the sample size in Experiment 2 was likely too small for a proper examination of correlational data with respect to statistical significance. Fourth, our groups were matched in terms of vocabulary (an issue that we noted as a limitation in prior research). The mean for the ASD group was 41.37 (SD = 7.73) and the mean for the control group was 40.09 (8.95). The difference was not significant *t* (39) = 0.49, *p* = 0.32. The results showed that vocabulary correlated significantly with non-modal cloze (see Table 8) and was a significant predictor when included in a regression model (see Table 9). In contrast, there were no significant correlations between the vocabulary and reaction times, and they were consistently negative (i.e., from −0.06 to −0.10).

### 3.3. Discussion

To summarize the main findings, the mean cloze probabilities were virtually identical in Experiment 1 and Experiment 2. However, we observed two significant effects with respect to the reaction times, which were consistent with our hypothesis that individuals with ASD would be slower, indicating worse linguistic prediction. The effect sizes for the significant results were medium, and the effect size for the non-significant result was small. In other words, in the online sentence completion measure, the reaction times for the selection of relevant and appropriate completions within each group were significantly different; thus, it took ASD individuals longer to produce equivalent-cloze-probability responses.

The results of the AQ correlational analysis were similar in many ways to the results of Experiment 1 and distinct in other ways. It is interesting to note that the communication subscale of the AQ produced the largest correlation (in Experiment 2) and it was for the overall cloze probability. The key difference across the two experiments was in the results for the modal responses, which were largely positive in Experiment 1 and largely negative in Experiment 2. We discuss these results further in Section 4.

## 4. General Discussion

The current study sought to investigate whether there is evidence for linguistic prediction deficits in ASD [6,12,50]. In an attempt to understand how the phenotypes of autism and its correlates can be explained, researchers have suggested that individuals with ASD have difficulty in predicting the dynamic world, and, therefore, manifestations of the disorder are a reflection of this clinically significant domain-general inability [8,10,11].

The current study specifically aimed to determine whether these prediction issues could be extended to a psycholinguistic paradigm using a cloze probability task (i.e., are deficits in prediction also observed in linguistic prediction?). The results, across both experiments, showed that there were no significant differences between the groups in the cloze probability of the words produced. This was not in line with our main predictions, perhaps suggesting that the deficits in prediction that have been observed in other studies are not domain-general. However, autistic traits, measured by the AQ [46], did have a significant effect on the non-modal cloze probabilities and marginal effects on the overall cloze probabilities and modal probabilities in Experiment 1. The effect on the modal probabilities showed that individuals with high AQ scores produced higher-cloze-probability responses (i.e., opposite to our prediction). The marginal effect of the AQ scores on the overall probabilities, and the significant effect of the AQ scores on the non-modal probabilities, suggests that, in cases where the modal response is not produced, individuals with higher levels of ASD traits produce significantly lower-cloze-probability responses. Thus, the evidence was mixed, with worse prediction in non-modal responses but the opposite for modal responses.

The assumption of the cloze task (and particularly for high-constraint items) is that one or two possible continuations are strongly activated, with perhaps three or four for less constraining items [44]. The finding of differences with non-modal items suggests that, in cases where the most probable word is not produced, the prediction essentially falls apart, and participants produce, in response, a much lower-probability word. Whatever the cause of the tendency to produce less probable non-modal responses, it is greater than the tendency to produce a higher rate of modal responses (i.e., more predictable responses). In other words, the non-modal effect is numerically greater/stronger than the reversed modal effect, as can be seen in the (negative) overall cloze probabilities. Note that this conclusion is based on the patterns of positive/negative results across the dependent variables, and we fully acknowledge that one of the results only achieved a marginally significant difference. The effect sizes of the AQ total on the cloze probabilities in Experiment 1 were small to medium.

In Experiment 2, we fully replicated the non-significant group effect of the cloze probability. However, consistent with the predictions, we observed that participants with ASD had slower reaction times, which, when averaged across all items, showed an approximately 200 ms mean delay in the voice onset time; this is consistent with a greater inefficiency in linguistic prediction in ASD. The effect sizes for the significant reaction time differences were medium. Another key difference between the studies was that AQ communication was significantly related to the overall cloze probabilities in Experiment 2 but not in Experiment 1. We concluded, based on the second set of regression analyses in Experiment 1, that the prediction differences associated with ASD traits were more likely due to the restricted interests/repetitive behaviours symptom domain, rather than social interaction and communication. We did not assess repetitive behaviours in Experiment 2 and so we were unable to run the analogous tests in Experiment 2. However, the difference between the experiments with respect to AQ communication warrants further investigation. To summarise the findings across both studies, we observed some evidence for weaker (and/or less efficient) linguistic prediction in ASD and in those with higher levels of ASD traits [33,34]. Furthermore, the analyses consistently demonstrated that the significant effects were not due to age or gender differences, and, in Experiment 2, our groups were matched in terms of vocabulary (cf. [35]).

To summarise, our study shows a mixed and relatively complex picture of the linguistic prediction issues as they relate to ASD traits. The findings for the group comparisons were more straightforward. Returning to the issues raised in the Introduction, and the beneficial aspects of prediction on (general) language comprehension abilities, we speculate that deficits and/or inefficiencies in linguistic prediction would necessarily make language comprehension more difficult. However, the issue of individual differences in prediction has not yet been explored in the psycholinguistic literature. Instead, psycholinguists have, for the past 10–15 years, focused on what is predicted (level of representation issues–semantic vs. phonological) and when (examining ERP measures and eye tracking). We hope that this study and others examining clinical populations contribute to the psycholinguistic literature to highlight individual differences in prediction and what may be the larger impacts of reduced linguistic prediction on other language processes.

### 4.1. Autism Diagnosis vs. Broad Autism Traits

One question arising from these results is how possessing a high level of autistic traits (perhaps possessing the broad autism phenotype) may result in predictive abilities that are in line with our hypotheses through the lens of the theory of predictive impairment in autism (PIA). We raise this issue given the differences between the group analysis and the AQ scores in Experiment 1. We also note that the correlations with repetitive behaviours were patterned similarly to the AQ scores but were actually larger. Thus, we tentatively concluded that the repetitive behaviour domain of ASD may be more strongly linked with weaknesses in predictive abilities, although more work is needed to confirm this result. Some researchers argue that there may be some merit to the idea that the theory of prediction is not apparent across all observed phenotypes of ASD. Cannon et al. [13] suggested that attempting to unify diverse aspects of ASD risks diminishing the complexity of the disorder. They suggest that the theory of prediction would instead better explain some correlates and traits of ASD, but should not be used as one unifying theory to encompass the whole disorder. Therefore, it is possible that the trends in the current study regarding linguistic prediction are an example of how correlations with autistic traits (i.e., the broad autism phenotype) are somewhat independent of a unifying and comprehensive account of prediction. Further research could extend this idea by identifying individuals with the broad autism phenotype with familial relations to individuals with ASD, to determine where the cut-off is for the “window of predictability” observed in the current study.

### 4.2. Limitations and Future Directions

The current study had several limitations. The first one is that we did not administer the repetitive behaviours questionnaire in Experiment 2. Given the strength of the correlations observed in Experiment 1, it would have been useful to corroborate these findings. Second, there were gender imbalances across the groups in both studies. The groups in Experiment 1 were significantly different and fell short of significance in Experiment 2. However, we did assess gender in regression models to ensure that the group differences remained. Third, the sample in Experiment 2 was quite limited and this prevented thorough conclusions for the correlational data; it also limits the complexity of the regression models, which can be run with any degree of confidence. (Note that this limitation applies to the data reported in Table 9.) We believed that it was important to report the correlations so that they could be compared to the results in Experiment 1; this provided another means to ensure/assess replicability. Fourth, we calculated cloze probabilities based on our entire sample of participants (including individuals with ASD), rather than calculating them based only on control data. In an earlier work [43], we found substantial differences compared to the current data and data reported in the original study [45], in which we obtained the sentence stems. In short, there are some clear differences (change over time) from the original data, which necessitate the calculation of cloze probabilities based on a modern cultural context. We did closely examine the words produced for each item by group and the differences were minimal, affecting less than 5% of the data. In addition, the rate of singleton responses was highly similar for ASD participants and controls (15% vs. 14%, respectively). Thus, we feel that combining the groups for the purpose of assessing the overall cloze probabilities did not impact the results reported here. Finally, there were some age effects on prediction, and, unfortunately, there were some age differences in our online study. One obvious future direction would be to place the participants under time pressure, requiring them to respond within a certain timeframe. Our prediction for such an experiment is that we would observe significant differences between the groups in the cloze probability (i.e., if we eliminated the reaction time differences between the groups, the result would be less effective prediction in ASD and hence lower-cloze-probability responses). We might also find an increased number of “I don’t know” responses in the ASD group.

## 5. Conclusions

The current study sought to investigate whether atypicalities in predictive abilities in ASD were present in across domains, namely in linguistic prediction. There are three main results of this study. First, there were small to medium effects of linguistic prediction. Small effects were observed in the offline measure in Experiment 1 on autism traits, and medium effects were observed in the online measure (reaction times) in Experiment 2. Second, in Experiment 2, we observed a medium-sized relationship between the overall cloze probability and the communication subscale of the AQ, with age and gender controlled. In contrast, in Experiment 1, repetitive behaviours were more related to the cloze probabilities compared to AQ communication. Third, the differences that we observed in terms of linguistic prediction align with domain-general assumptions of weakness in prediction, although, clearly, more work is needed to definitively support this conclusion. Given these results, we believe that the main impact of inefficiency in linguistic prediction relates to language comprehension more generally and the fact that it would make language comprehension more difficult overall.

## Figures and Tables

**Table 1:** Means and inferential tests for demographic variables, AQ scores, and repetitive behaviours.

	ASD (33)	Control (64)	Significance (Cohen’s D)
Variable	Mean (SD)	Mean (SD)	
Demographic Variables			
Age	30.76 (12.31)	34.03 (12.30)	*t* (95) = −1.24, *p* = 0.22 (−0.27)
Gender (% male)	15.2	23.4	*t* (95) = 2.78, *p* < 0.01 (0.60)
Autism Quotient			
AQ Social Skills	7.12 (1.71)	2.94 (2.08)	*t* (95) = 9.96, *p* < 0.001 (2.13)
AQ Attention Switching	9.06 (1.00)	4.42 (2.27)	*t* (95) = 11.19, *p* < 0.001 (2.40)
AQ Attention Details	7.30 (2.17)	5.39 (2.24)	*t* (95) = 4.02, *p* < 0.001 (0.86)
AQ Communication	7.52 (2.09)	2.38 (2.03)	*t* (95) = 11.70, *p* < 0.001 (2.51)
AQ Imagination	5.33 (2.07)	2.48 (1.60)	*t* (95) = 7.51, *p* < 0.001 (1.61)
AQ TOTAL	36.33 (5.88)	17.61 (7.00)	*t* (95) = 13.16, *p* < 0.001 (2.82)
Repetitive Behaviours			
ARBQ	38.61 (7.01)	27.08 (5.80)	*t* (95) = 8.63, *p* < 0.001 (1.84)

Note. ASD = autism spectrum disorder, SD = standard deviation, AQ = Autism Quotient, ARBQ = Adult Repetitive Behaviours Questionnaire.

**Table 2:** Example stimuli, with cloze probabilities from Arcuri et al. [45].

In the distance, they heard the _______.
*Cloze Probabilities:* noise (0.32), thunder (0.10), birds (0.06), scream (0.06)
The hunter shot a large _______.
*Cloze Probabilities:* deer (0.78), lion (0.06), bear (0.06)
John wisely chose to pay the _______.
*Cloze Probabilities:* bill (0.50), debt (0.08), taxes (0.04), man (0.04)
At night, the old woman locked the _______.
*Cloze Probabilities:* door (0.76), basement (0.04)

**Table 3:** Results of independent-samples *t*-tests.

	ASD	Control	Significance ^1^ (Cohen’s D)
	Mean (SD)	Mean (SD)	Mean (SD)
Overall Cloze	0.36 (0.06)	0.37 (0.07)	*t* (95) = −0.77, *p* = 0.22 (−0.16)
Modal	0.60 (0.03)	0.59 (0.04)	*t* (95) = 1.33, *p* = 0.09 (0.28)
Non-Modal	0.10 (0.02)	0.11 (0.03)	*t* (94) = −0.77, *p* = 0.22 (−0.17)

Note. ^1^ Significance is reported at the one-tailed level. ASD = autism spectrum disorder, SD = standard deviation.

**Table 4:** Results of Kendall’s Tau correlations, with *p*-values in parentheses following each correlation.

	Overall	Modal CP	Non-Modal CP
Age	0.02 (0.76)	−0.14 (0.04) *	0.01 (0.88)
Gender	0.28 (<0.001) *	−0.03 (0.75)	0.17 (0.04)
AQ total	−0.13 (0.06)	0.07 (0.30)	−0.14 (0.04)
Symptom Domains			
AQ Communication	−0.16 (0.03) *	0.06 (0.40)	−0.14 (0.06)
ARBQ Repetitive	−0.21 (0.003) *	0.19 (0.006) *	−0.18 (0.01) *

Note. * indicates significant result following false discovery rate correction. Gender was coded as 0 = male and 1 = female. Other/unreported genders were omitted from the analysis. CP = cloze probability, AQ = Autism Quotient, ARBQ = Adult Repetitive Behaviours Questionnaire.

**Table 5:** Multiple regression results and coefficients.

Variable	B	SE (B)		β	*t*-Value (*p*-Value)	CILB	CIUB
1. Overall High F (3,93) = 3.60, *p* < 0.05, R^2^ = 0.10							
Age	0.000	0.001	−0.084	−0.854 (0.395)	−0.000	0.001	
Gender		0.035	0.013	0.279	2.81 (0.006)	0.010	0.061
AQ Total	−0.001	0.001	−0.193	−1.94 (0.056)	−0.002	0.000	
2. High Modal F (3,93) = 3.91, *p* < 0.05, R^2^ = 0.11							
Age	−0.001	0.000	−0.272	−2.78 (0.007)	−0.001	0.000	
Gender		−0.003	0.007	−0.039	−0.391 (0.697)	−0.017	0.011
AQ Total	0.001	0.000	0.192	1.94 (0.055)	0.000	0.001	
3. High Non-Modal F (3,92) = 3.04, *p* < 0.05, R^2^ = 0.09							
Age	0.000	0.000	0.024	0.245 (0.807)	0.000	0.001	
Gender		0.012	0.006	0.224	2.22 (0.029)	0.001	0.023
AQ Total	−0.001	0.000	−0.239	−2.37 (0.020)	−0.001	0.000	
4. Overall High F (4,92) = 3.67, *p* < 0.05, R^2^ = 0.13							
Age	−0.001	0.001	−0.122	−1.23 (0.227)	−0.002	0.000	
Gender		0.034	0.013	0.267	2.58 (0.011)	0.008	0.060
AQ Communication	−0.004	0.003	−0.174	−1.19 (0.235)	−0.009	0.002	
ARBQ Repetitive	−0.001	0.001	−0.094	−0.633 (0.528)	−0.003	0.002	
5. High Modal F (4,92) = 3.71, *p* < 0.05, R^2^ = 0.14							
Age	−0.001	0.000	−0.210	−2.09 (0.039)	−0.001	0.000	
Gender		0.003	0.007	0.042	0.412 (0.681)	−0.011	0.017
AQ Communication	−0.001	0.002	−0.119	−0.823 (0.413)	−0.005	0.002	
ARBQ Repetitive	0.001	0.001	0.334	2.28 (0.025)	0.000	0.003	
5. High Non-Modal F (4,91) = 2.59, *p* < 0.05, R^2^ = 0.10							
Age	−0.000	0.000	−0.031	−0.306 (0.760)	−0.001	0.000	
Gender		0.010	0.006	0.175	1.66 (0.100)	−0.002	0.021
AQ Communication	−0.001	0.001	−0.065	−0.442 (0.659)	−0.003	0.002	
ARBQ Repetitive	−0.001	0.001	−0.216	−1.44 (0.152)	−0.002	0.000	

Note. SE = standard error, CILB = confidence interval lower bound, CIUB = confidence interval upper bound, AQ = Autism Quotient, ARBQ = Adult Repetitive Behaviours Questionnaire.

**Table 6:** Means and inferential tests for demographic variables and AQ scores.

	ASD (19)	Control (22)	Significance (Cohen’s D)
Variable	Mean (SD)	Mean (SD)	
Demographics			
Age	20.0 (1.83)	19.91 (2.52)	*t* (39) = −0.13, *p* = 0.98 (0.04)
Gender (% male)	47.4	22.7	*t* (36) = 1.89, *p* = 0.07 (0.62)
Autism Quotient			
AQ Social Skills	6.47 (1.83)	2.41 (1.79)	*t* (39) = 7.23, *p* < 0.001 (2.28)
AQ Attention Switching	8.89 (1.20)	5.32 (2.06)	*t* (39) = 6.66, *p* < 0.001 (2.09)
AQ Attention Details	7.05 (2.12)	5.73 (2.55)	*t* (39) = 1.79, *p* = 0.08 (0.56)
AQ Communication	7.84 (1.98)	3.05 (2.40)	*t* (39) = 6.91, *p* < 0.001 (2.17)
AQ Imagination	5.16 (2.22)	2.23 (1.60)	*t* (39) = 4.90, *p* < 0.001 (1.53)
AQ TOTAL	35.42 (6.97)	18.73 (6.16)	*t* (39) = 8.14, *p* < 0.001 (2.56)

Note. Two participants with ASD reported a non-binary gender and one control reported an “other” gender. These participants were not included in the gender analysis. ASD = Autism Spectrum Disorder, SD = standard deviation, AQ = Autism Quotient.

**Table 7:** Results of independent-samples *t*-tests for cloze probability and reaction time. Effect size (Cohen’s D) in parentheses following each *p*-value.

	ASD	Control	Significance (Cohen’s D) ^1^
	Mean (SD)	Mean (SD)	
Overall Cloze	0.36 (0.04)	0.37 (0.04)	*t* (39) = −1.03, *p* = 0.16 (−0.32)
Reaction Time	1144 (478)	923 (206)	*t* (39) = 1.71, *p* = 0.05 (0.54)
Modal	0.60 (0.03)	0.61 (0.02)	*t* (39) = −1.32, *p* = 0.10 (−1.03)
Reaction Time	957 (439)	727 (155)	*t* (39) = 2.06, *p* = 0.02 (0.65)
Non-Modal	0.10 (0.02)	0.09 (0.02)	*t* (39) = 0.66, *p* = 0.26 (0.21)
Reaction Time	1353 (547)	1187 (352)	*t* (39) = 0.88, *p* = 0.19 (0.28)

Note. ^1^ Significance is reported at the one-tailed level. ASD = Autism Spectrum Disorder, SD = standard deviation.

**Table 8:** Results of Kendall’s Tau correlations, with *p*-values in parentheses following each correlation.

	Overall CP	Modal CP	Non-Modal CP
Age	−0.11 (0.34)	−0.05 (0.66)	−0.27 (0.02) *
Gender	−0.14 (0.29)	0.06 (0.67)	−0.12 (0.36)
AQ total	−0.12 (0.27)	−0.12 (0.27)	−0.07 (0.55)
PPVT	0.19 (0.08)	−0.19 (0.08)	0.37 (<0.001) *
AQ Subscale			
Communication	−0.27 (0.02)	−0.10 (0.36)	−0.14 (0.20)

Note. * indicates significant result following false discovery rate correction. Gender was coded as 0 = male and 1 = female. Other/unreported genders were omitted from the analysis. CP = cloze probability, AQ = Autism Quotient, PPVT = Peabody Picture Vocabulary Test.

**Table 9:** Multiple regression results and coefficients.

Variable	B	SE (B)	β	*t*-Value (*p*-Value)	CILB	CIUB
1. Overall High F (4,33) = 3.79, *p* < 0.05, R^2^ = 0.32						
Age	−0.004	0.003	−0.23	−1.52 (0.138)	−0.009	0.001
Gender	0.013	0.013	0.159	1.00 (0.323)	−0.013	0.039
AQ Communication	−0.005	0.002	−0.427	−2.87 (0.007)	−0.009	−0.002
PPVT	0.001	0.001	0.243	1.591 (0.121)	0.000	0.003
2. High Modal F (4,33) = 0.627, *p* = 0.65, R^2^ = 0.07						
Age	0.002	0.002	0.131	0.754 (0.456)	−0.003	0.006
Gender	−0.002	0.010	−0.041	−0.219 (0.828)	−0.024	0.019
AQ Communication	0.000	0.002	−0.015	−0.089 (0.929)	−0.003	0.003
PPVT	−0.001	0.001	−0.233	−1.31 (0.200)	−0.002	0.000
3. High Non-Modal F (4,33) = 3.41, *p* < 0.05, R^2^ = 0.29						
Age	−0.002	0.001	−0.158	−1.04 (0.304)	−0.005	0.001
Gender	0.001	0.007	0.027	0.165 (0.870)	−0.014	0.016
AQ Communication	−0.001	0.001	−0.071	−0.473 (0.639)	−0.003	0.002
PPVT	−0.001	0.000	−0.518	3.40 (0.002)	0.001	0.002

Note. SE = standard error, CILB = confidence interval lower bound, CIUB = confidence interval upper bound, AQ = Autism Quotient, PPVT = Peabody Picture Vocabulary Test.

## Data Availability

The raw data supporting the conclusions of this article will be made available by the authors on request.
